# Effect of Calcium Hydroxy-Methyl-Butyrate-Enriched Diabetes-Specific Oral Nutritional Supplementation on Patients with Heterogeneous Diabetes Mellitus Population with Disease Related Malnutrition Assessed with AI-Assisted Ultrasound Imaging

**DOI:** 10.3390/nu17203208

**Published:** 2025-10-13

**Authors:** Juan J. López-Gómez, Jaime González-Gutiérrez, Paloma Pérez-López, Olatz Izaola-Jauregui, Ángela Cebriá, Lucía Estévez-Asensio, David Primo-Martín, Mario Alfredo Saavedra-Vasquez, Beatriz Ramos-Bachiller, Daniel Rico-Bargues, Eduardo Jorge Godoy, Daniel Antonio De Luis-Román

**Affiliations:** 1 Servicio de Endocrinología y Nutrición, Hospital Clínico Universitario de Valladolid, 47003 Valladolid, Spainlesteveza@saludcastillayleon.es (L.E.-A.); dprimoma@saludcastillayleon.es (D.P.-M.); masaavedra@saludcastillayleon.es (M.A.S.-V.); dadluis@yahoo.es (D.A.D.L.-R.); 2 Centro de Investigación en Endocrinología y Nutrición, Universidad de Valladolid, 47003 Valladolid, Spain; daniel.rico@uva.es; 3 Health Research Institute of Valladolid (IBioVALL), 47010 Valladolid, Spain; 4 Dawako Medtech S.L., Parc Cientific de la Universitat de Valencia, 46980 Paterna, Spain; acebria@dawako.es (Á.C.); egodoy@dawako.es (E.J.G.); 5 Técnicas Avanzadas de Desarrollo de Software Centrado en la Persona, Departamento de Informática, Universitat de Valencia, 46010 Valencia, Spain

**Keywords:** beta hydroxy-methyl-butyrate, muscle ultrasound, artificial intelligence, diabetes mellitus, disease-related malnutrition

## Abstract

**Background/Objectives**: Sarcopenia is common in patients with diabetes mellitus. The use of branched-chain amino acids may influence muscle mass. The aim of this study is to evaluate the effect of a diabetes-specific formula enriched with calcium hydroxy-methyl-butyrate (CaHMB) on muscle mass in patients with diabetes and high risk of malnutrition. **Methods**: A prospective observational study in 95 patients divided into two cohorts of patients with diabetes, treated with a tailored diet, dietary counseling, and diabetes-specific oral nutritional supplements (ONSs) administered between meals: one enriched with CaHMB (CaHMB Diabetes ONS) 44 (46.32%) patients; and another without CaHMB (Diabetes-Specific ONS) 51 (53.68%) patients. Anthropometric parameters, bioimpedance, artificial intelligence (AI)-assisted ultrasound of the rectus femoris muscle (PIIXMED^TM^), and handgrip strength were assessed. Evaluations were conducted at baseline and after 3 months. **Results**: The mean age was 71.05 (10.67) years; 56.8% were male. After three months, both groups increased their nutritional intake with no differences in dietary protein content between groups. The CaHMB group showed a greater increase in muscle mass as measured by ultrasound, both in muscle area (CaHMB ONS: +5.84 (−3.3 ± 21.58)% vs. Diabetes-Specific ONS: −9.34% (−25.78 ± 12.02)%; *p* < 0.01) and muscle thickness (CaHMB ONS: +9.17 (−4.40 ± 21.05)% vs. Diabetes-Specific ONS −6.30 (−18.57 ± 12.56)%; *p* < 0.01). The CaHMB ONS group showed a higher likelihood of increased muscle mass compared to the Diabetes-Specific ONS, with an odds ratio (OR) of 9.31 (95%CI: 2.16–40.13) for thickness and 3.96 (95%CI: 1.11–14.13) for area, adjusted for gender, age, serum albumin, and baseline glycated hemoglobin. **Conclusions**: Supplementation with Ca-HMB in patients with diabetes and high risk of malnutrition showed significant improvements in muscle mass as assessed by AI-assisted ultrasound. Both groups increased nutritional intake, but only the CaHMB group showed specific benefits in muscle parameters.

## 1. Introduction

Diabetes mellitus (DM) is a chronic disease affecting about 588.7 million adults worldwide, roughly 11.1% percent of the global adult population. Its prevalence is relatively low in younger age groups but rises steeply with age, reaching 24.8% among 75–79 year olds in 2024 [1]. People with DM face a markedly increased risk of nutritional problems. In elderly inpatients with T2DM, 39.1% are identified as at nutritional risk and 21.2% as malnourished according to the Mini Nutritional Assessment (MNA) [2]. Using the Global Leadership Initiative on Malnutrition (GLIM) criteria, the VIDA study found moderate malnutrition in 35.8% and severe malnutrition in 16.3% of this population [3]. The Semana de la Desnutrición Relacionada con la Enfermedad (SeDREno) study similarly reported that 29.7% of hospitalized DM patients met GLIM’s thresholds for disease-related malnutrition (12.5% severe, 17.2% moderate), with the rate rising to 34.8% among those aged 70 or older [4].

DRM is marked by a decrease in muscle mass, which serves as one of its diagnostic criteria [5], and this criterion may be present in 66.9% of patients diagnosed with DRM according to the GLIM criteria [6]. Sarcopenia is a condition characterized by reduced muscle mass and strength, often linked to aging, some chronic diseases, and DRM itself [7,8]. Diabetes can be linked to muscle atrophy through diabetic sarcopenia, an underdiagnosed complication arising from an imbalance between muscle protein synthesis and breakdown. In this condition, inflammation, oxidative stress, neuropathy, vasculopathy, insulin resistance, and antidiabetic drugs act as triggers [9]. This condition is independent of occurring either in DRM patients or in patients with obesity, and it is related to poor glycaemic control, duration, and presence of complications of DM [10]. The coexistence of disease-related malnutrition (DRM) and diabetes significantly accelerates muscle deterioration. Although the overall prevalence of malnutrition was similar in patients with and without diabetes, diabetes substantially amplified sarcopenia’s impact, underscoring the need for early nutritional assessment and targeted muscle-preserving interventions [11].

Patients with DRM require medical nutrition therapy based on an adapted diet and, when necessary, artificial nutrition tailored to their energy and protein requirements [12]. In patients with DM, dietary and artificial nutrition regimens must be specifically tailored to their unique metabolic profiles and glycaemic control requirements. Artificial nutrition, especially enteral nutrition, features low-glycaemic index carbohydrates, a specialized fiber blend rich in insoluble fiber, and, depending on the formula, a high lipid content with a monounsaturated fatty acid (MUFA) profile, particularly omega-9 [13]. Diabetes-specific formulas (oral supplements and tube feeds) have been shown to improve glycaemic control and metabolic risk profiles in patients with diabetes or stress-induced hyperglycemia compared with standard formulas [13]. Incorporating these formulas as oral nutritional supplements (ONS) alongside an adapted diet has been shown to reduce malnutrition rates in patients with carbohydrate metabolism disorders, with a 2022 study reporting a decrease from 80% to 51.7% [14]. The type of protein used in ONS, particularly branched-chain amino acids (BCAAs) such as leucine and its metabolite β-hydroxy-β-methylbutyrate (HMB), has the potential to enhance muscle mass and quality in patients with sarcopenia. This effect has been demonstrated in the meta-analysis by Geng-Hao Bai et al., which showed improvements in muscle mass and strength [15]. Additionally, Cogo et al. reported similar benefits in oncological surgical patients, with reduced postoperative morbidity compared to controls [16]. CaHMB-enriched oral nutritional supplements (ONS) have also improved nutritional status, muscle mass, and muscle quality, assessed via an artificial intelligence (AI) system, in patients at risk of malnutrition in the study by De Luis et al. [17]. Moreover, this type of ONS reduced mortality and enhanced nutritional status markers over a 90-day follow-up in the “Nutrition effect on unplanned Readmissions and Survival in Hospitalized patients” (NOURISH) study [18]. On the other hand, CaHMB-enriched supplements, especially combined with amino acids like lysine and arginine, have been shown in cellular and diabetes rodent models to increase GLUT4 expression and Akt signaling, enhance glucose uptake, improve insulin sensitivity, and preserve lean mass [19]. These complementary effects support using CaHMB-based formulations to target both muscle wasting and metabolic dysregulation in diabetes.

Considering the prevalence of DRM in the context of DM and the risk of sarcopenia associated with these two conditions, the use of targeted medical nutritional therapy may offer an added benefit to the patient’s nutritional rehabilitation. The administration of a Diabetes-Specific ONS enriched with CaHMB may exert a beneficial effect on the nutritional management of malnutrition, help improve metabolic control, and, through the action of BCAA metabolites, enhance muscle health in these patients. The purpose of this study was to evaluate the effect of a CaHMB-enriched oral nutritional supplement on nutritional status and muscle health in patients with diabetes mellitus at risk of malnutrition. The primary objective is to compare the effect of a CaHMB-enriched diabetes-specific formula versus a historical cohort of patients treated with a non-CaHMB-enriched diabetes specific formula on muscle mass, quality, and function.

## 2. Materials and Methods

### 2.1. Study Design and Eligibility Criteria

This prospective observational study analyzed two cohorts of diabetic patients at risk of malnutrition who received personalized nutritional interventions. One cohort was treated with an adapted diet combined with a diabetes-specific oral nutritional supplement enriched with calcium β-hydroxy-β-methylbutyrate (CaHMB Diabetes ONS), while the historical cohort received an adapted diet alongside a hypercaloric high-protein oral nutritional supplement (Diabetes-Specific ONS) without CaHMB. Participants were recruited from the Clinical Nutrition Unit of the University Clinical Hospital of Valladolid between January 2021 and March 2025.

Each patient underwent a comprehensive interview covering medical history, disease progression, and nutritional status. Nutritional assessments included anthropometric measurements, bioelectrical impedance analysis (BIA), muscle ultrasonography, and evaluations of muscle strength. Biochemical parameters related to nutritional status, inflammation, and metabolism were also collected. Assessments were conducted at baseline and repeated three months after the initiation of treatment.

All patients were screened for malnutrition risk using the Malnutrition Universal Screening Tool (MUST) [20], and diabetes was diagnosed according American Diabetes Association (ADA) criteria in previously undiagnosed individuals [21]; patients with a prior diagnosis were identified by the presence of antidiabetic treatment, evaluating the pattern of the history of diabetes in clinical records to evaluate the type of diabetes. The study included individuals aged 18 years and older. Exclusion criteria comprised neurological disorders with inability to ambulate, chronic corticosteroid treatment, chronic kidney disease at stage IV or above, uncontrolled liver disease, oncologic patients in palliative care without any oncological treatment, and refusal to provide informed consent.

The study was approved by the Medical Research Ethics Committee (CEIm) of the Valladolid Health Areas (approval code: PI-24-488-C, date of approval: 24 July 2024). All procedures adhered to the ethical principles set forth in the Declaration of Helsinki. Written informed consent was obtained from each eligible participant prior to enrolment.

### 2.2. Nutritional Intervention

The intervention consisted of a three-month nutritional plan combining dietary counseling and oral nutritional supplementation (ONS). Dietary counseling was provided by a qualified dietitian, who offered tailored advice strategies to enhance the nutritional quality of the patients’ regular diet. In addition to dietary guidance, a physician prescribed an ONS to increase caloric and protein intake.

Participants were allocated by consecutive sampling into two groups: a historical cohort receiving a hypercaloric, high-protein, diabetes-specific oral nutritional supplement (ONS) formulated with low-glycaemic index carbohydrates and enriched in monounsaturated fatty acids and fiber (Diabetes-Specific ONS); and an intervention group receiving a diabetes-specific ONS with the same qualitative composition, additionally enriched with calcium β-hydroxy-β-methylbutyrate (CaHMB Diabetes ONS). The nutrient composition of each supplement is detailed in Table 1.

The prescribed dosage was individualized according to each patient’s nutritional requirements and dietary intake, with prescriptions ranging from one to two bottles per day.

### 2.3. Study Variables

The study variables included age (expressed in years), gender (male/female), and the presence of pathologies associated with malnutrition.

#### 2.3.1. Body Composition and Muscle Function

Anthropometry: Anthropometric measurements included height (m), weight (kg), and body mass index (BMI, kg/m^2^). Weight was recorded unclothed on a precision scale (+0.1 kg; Seca, Birmingham, UK), and height was measured in a standing position using a stadiometer (Seca, Birmingham, UK). BMI was calculated as weight divided by height squared. Percentage weight loss was determined by the formula [(Usual weight (kg) − Current weight (kg))/Usual weight] × 100. Upper arm circumference was measured on the right arm at the midpoint between the acromion and olecranon processes, with the arm relaxed alongside the torso. Calf circumference was measured on the right leg at its greatest girth while the participant was seated, knees bent at 90°, feet flat on the floor. Both circumferences were recorded in centimeters to the nearest 0.1 cm using a non-stretchable tape.

Bioelectrical impedance analysis (BIA): This technique was performed using a NUTRILAB device (EFG Akern, Milan, Italy) delivering a 0.8 mA alternating current at 50 kHz. After at least a 5-h fast, electrodes were placed on the dorsal surfaces of the right hand and foot to measure reactance (Xc), resistance (Rz), and phase angle. Appendicular muscle index (ASMI) was then calculated using Sergi’s formula [22].

Artificial intelligence-assisted muscle ultrasonography: Muscle ultrasonography of the quadriceps rectus femoris (QRF) on the dominant leg was performed with a 10–12 MHz linear-array probe (Mindray Z60, Shenzhen, China). The probe was placed perpendicular to the transverse axis at the distal third of the distance between the anterior iliac crest and the superior patellar border, with the patient in a supine position [23]. All scans were acquired by a single, trained operator following a standardized protocol-applying minimal pressure to avoid tissue deformation and ensure consistent image quality.

**Figure 1:**
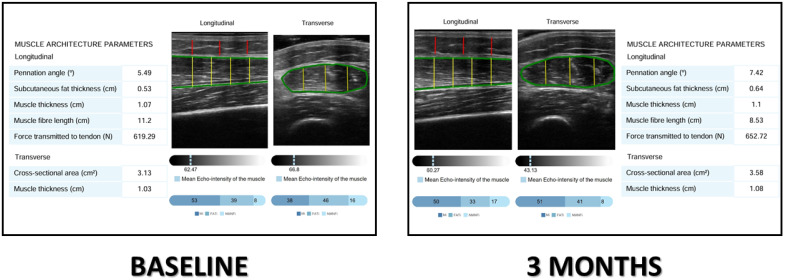
Image analysis by PIIXMED^TM^ in a patient before and after the treatment. Mi: muscle index; FATi: fat index; NMNFi: no muscle, no fat index. Lines: Green: rectus femoris fascia, red: anteroposterior adipose tissue thickness; yellow: anteroposterior axis of muscle.

Images were processed using an AI-based system (PIIXMED^TM^ MSK; Dawako Medtech, S.L., Valencia, Spain), which automatically extracted B-mode features within a defined region of interest (ROI) and applied algorithms to derive biomarkers of muscle morphology, echogenicity, and texture. Muscle mass was quantified by rectus femoris cross-sectional area (RFMA, cm^2^) and muscle thickness (RFMT, cm), while subcutaneous fat thickness (SFT, cm) was measured in the longitudinal plane. Muscle quality was assessed by pennation angle, the angle between muscle fibers and the deep aponeurosis, and by three grayscale-based indices generated via Multi-Otsu thresholding: the muscle index (Mi), fat index (FATi), and no muscle no fat index (NMNFi), each expressed as a percentage of the ROI in the cross-sectional plane [24]. This segmentation method has demonstrated excellent agreement with manual analysis, with intraclass correlation coefficients exceeding 0.90 for all parameters [25]. Another quality parameter used in cross-sectional planes was the Y/X index, where the Y index is the RFMT and X is the transversal measure; the predominance of muscle thickness against transversal diameter is considered related to better functionality of muscle [26] (Figure 1).

Muscle function: It was evaluated by measuring handgrip strength of the dominant hand with a JAMAR^TM^ hydraulic dynamometer. Participants were seated with the elbow flexed at 90° and the forearm in a neutral position. Three trials were performed, allowing a brief rest between attempts, and the highest value was recorded.

#### 2.3.2. Biochemical Variables

Biochemical variables were analyzed using a Cobas c711 autoanalyzer (Roche Diagnostics, Basel, Switzerland). The nutritional and inflammation parameters include albumin, measured in grams per deciliter (g/dL); C-Reactive Protein (CRP), measured in milligrams per deciliter (mg/dL); prealbumin, also in mg/dL; and the CRP/prealbumin ratio, which provides insight into the balance between inflammatory response and nutritional status. The metabolic parameters assessed include glucose measured in milligrams per deciliter (mg/dL); total cholesterol (mg/dL); high-density lipoprotein (HDL) cholesterol (mg/dL); low-density lipoprotein (LDL) cholesterol (mg/dL); triglycerides (mg/dL); and glycated hemoglobin (HbA1c), expressed as a percentage (%), which reflects long-term glycaemic control.

#### 2.3.3. Nutritional Survey

All participants completed a 2-day prospective dietary record to assess energy and macronutrient intake. The survey was administered at baseline (before the intervention) and again at 3 months. Participants were instructed not to alter their usual eating patterns to ensure representativeness, and to record all foods and beverages daily using kitchen scales to improve portion-size accuracy; preparation methods were also documented. Records were reviewed by a dietitian and analyzed using Dietsource (Nestlé, Geneva, Switzerland). Total energy intake (kcal) was used as a primary indicator of dietary intake. Nutrient intake was quantified both as absolute amounts (kcal or g) and as percentages of total energy; macronutrients included protein, carbohydrates, fat, and fiber (all in grams per day). Macronutrients were expressed in grams and percentage of total caloric value (%TCV). The analysis also included total lipids and the fatty acid profile (monounsaturated (MUFA), polyunsaturated (PUFA), and saturated fatty acids (SFA), as well as dietary cholesterol), each expressed in grams per day.

#### 2.3.4. Nutritional Diagnosis

Malnutrition was defined according to the Global Leadership Initiative on Malnutrition (GLIM) criteria, which require at least one phenotypic and one etiologic criterion [27]. Sarcopenia was assessed following the European Working Group on Sarcopenia in Older People (EWGSOP2): low muscle strength was defined by low handgrip strength (<27 kg in men and <16 kg in women) and reduced muscle quantity defined by BIA-estimated Appendicular Skeletal Muscle Index (ASMI) (Appendicular Skeletal Muscle Index assessed by BIA as <7 kg/m^2^ in men and <5.5 kg/m^2^ in women) [28]. Dynapenia was defined as only low muscle strength without low muscle mass.

### 2.4. Data Analysis

Statistical analyses were performed with SPSS version 23.0 (SPSS Inc., Chicago, IL, USA) under license from the University of Valladolid. Continuous variables were tested for normality using the Kolmogorov–Smirnov test and reported as mean (Standard Deviation) or median (p25–p75), as appropriate.

Parametric differences were assessed by paired or independent-sample Student’s *t*-tests. Nonparametric comparisons used the Wilcoxon signed-rank test for paired data, the Mann–Whitney U test for two independent groups, the Kruskal–Wallis test for more than two independent groups, and the Friedman test for repeated measures. When comparing more than two groups under parametric assumptions, one-way ANOVA with Bonferroni post hoc adjustment was applied. Changes across time points were also evaluated by repeated-measures ANOVA. Categorical variables are expressed as frequencies and percentages and compared using the chi-square test, with Fisher’s exact test or Yates’ continuity correction when cell sizes were small.

Multivariate analysis with logistic regression was used to estimate the odds of body composition improvement, defined as a percentage change from baseline exceeding zero, with supplement type as the primary predictor (CaHMB Diabetes ONS = 1; Diabetes-Specific ONS = 0). To mitigate potential confounding, all models were adjusted for gender, age, serum albumin, and HbA1c. Statistical significance was defined as a *p*-value < 0.05.

## 3. Results

### 3.1. Sample Description

**Figure 2:**
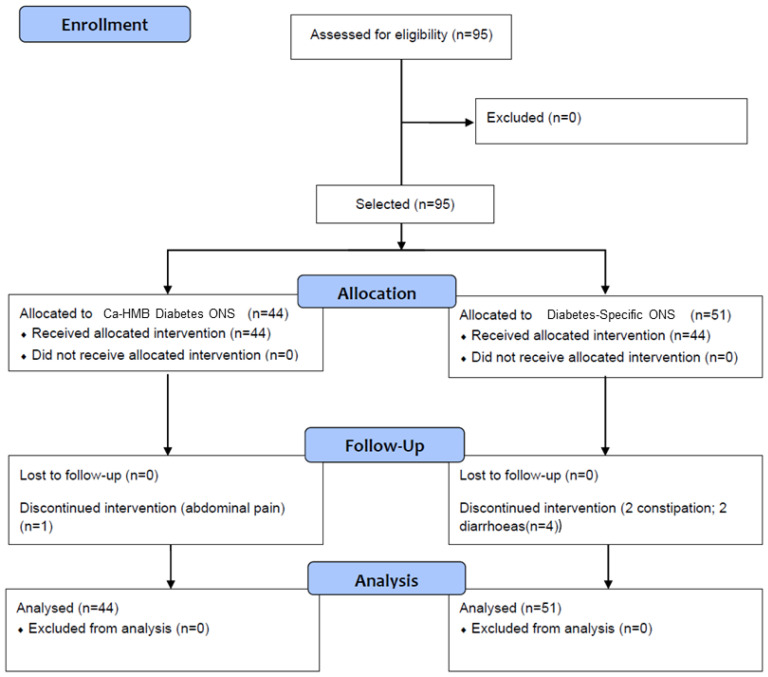
Flow chart of enrollment, allocation, and follow-up of patients. Hydroxy-methyl-butyrate-enriched diabetes-specific oral nutritional supplementation (CaHMB Diabetes ONS) vs. Historic Cohort Supplements (Diabetes-Specific ONS).

A total of 95 patients with diabetes mellitus at risk of malnutrition were recruited. Of these, 51 patients (53.7%) were assigned to the control group (historic cohort receiving the standard diabetes-specific oral nutritional supplement (Diabetes-Specific ONS), and 44 patients (46.3%) were assigned to the intervention group (receiving a diet supplemented with hydroxy-methyl-butyrate-enriched diabetes specific oral nutritional supplement (CaHMB Diabetes ONS)). All patients completed follow-up; however, 5 patients (5.26%) discontinued the intervention and required a change to a different diabetes-specific oral nutritional formula—1 patient (2.27%) from the CaHMB Diabetes ONS group and 4 patients (7.8%) from the Diabetes-Specific ONS group (Figure 2).

The mean age of patients was 71.05 (10.67) years, and 54 patients (56.8%) were male. Most patients had oncological disease (57 (60%)), without differences between groups (CaHMB Diabetes ONS: 26 (59.1%); Diabetes-Specific ONS: 31 (60.8%)). All patients had diabetes mellitus (Type 1 DM: 7 (7.4%); Type 2 DM: 86 (83.2%); Type 3C DM: 8 (8.4%); Monogenic DM: 1 (1.1%), with no differences between groups (CaHMB Diabetes ONS: Type 1 DM: 4 (9.1%); Type 2 DM: 37 (84.1%); Type 3C DM: 3 (6.8); Diabetes-Specific ONS: Type 1 DM: 3 (5.9%); Type 2 DM: 42 (82.4%); Type 3C DM: 5 (9.8%); Monogenic DM: 1 (2%); (*p* = 0.69)). A total of 34 (35.8%) patients were treated with insulin, with no significant differences between groups (CaHMB Diabetes ONS: 12 (27.3%) patients; Diabetes-Specific ONS: 22 (43.1%) patients; *p* = 0.11).

A total of 78 patients (82.1%) were diagnosed with malnutrition, of whom 13 (13.7%) had severe malnutrition. Sarcopenia was present in 33 patients (34.7%), with no significant differences between groups (Table 2). No differences were observed in anthropometric measurements, body composition, or muscle strength between groups (Table 2). However, patients in the CaHMB Diabetes ONS group showed higher levels of serum albumin and HbA1c compared to the Diabetes-Specific ONS group (Table 2).

### 3.2. Changes in Diet Characteristics Before and After Intervention

Analysis prior to the intervention showed that patients consumed an average of 1393 (419) kcal per day. Mean protein intake was 66.65 (20.51) g/day, accounting for 20.17 (4.52) % of total caloric value (TCV); carbohydrate intake averaged 62.25 (22.95) g/day (42.57 (6.94) % TCV); and lipid intake was 62.25 (22.95) g/day (40.46 (8.62) % TCV). No significant differences were observed between intervention groups in terms of energy or macronutrient intake prior to the intervention.

Regarding the qualitative composition of dietary intake, the mean fiber consumption was 13.41 (5.27) g per day, with no significant differences between groups. In terms of dietary lipid intake, the CaHMB Diabetes ONS group showed higher consumption of saturated fatty acids (SFA) (CaHMB Diabetes ONS: 20.08 (8.82) g/day; Diabetes-Specific ONS: 16.28 (6.87); *p* = 0.04), monounsaturated fatty acids (MUFA) (CaHMB Diabetes ONS: 27.67 (9.68) g/day; Diabetes-Specific ONS: 22.61 (7.69) g/day; *p* = 0.01), and polyunsaturated fatty acids (PUFA) (CaHMB Diabetes ONS: 8.94 (4.80) g/day; Diabetes-Specific ONS: 5.55 (3.80) g/day; *p* < 0.01). No significant differences were observed in dietary cholesterol intake between the groups (CaHMB Diabetes ONS: 298 (138) mg/day; Diabetes-Specific ONS: 307 (135) mg/day; *p* = 0.78). The mean volume of ONS consumed in the total sample was 331.63 (117.55) mL. A slight increase was observed in the volume of ONS consumed with CaHMB Diabetes ONS 332.50 (119.77) mL/day compared to Diabetes-Specific ONS 116.79 (16.35) mL/day; *p* = 0.01. The mean intake of calories and protein from the ONS was significantly higher (*p* < 0.01) in the CaHMB group (Calories: 532 (191.63) kcal/day; Protein: 106.40 (38.33) g/day) compared Diabetes-Specific ONS group (Calories: 397.06 (140.15) kcal/day; Protein:87.35 (30.83) g/day).

**Figure 3:**
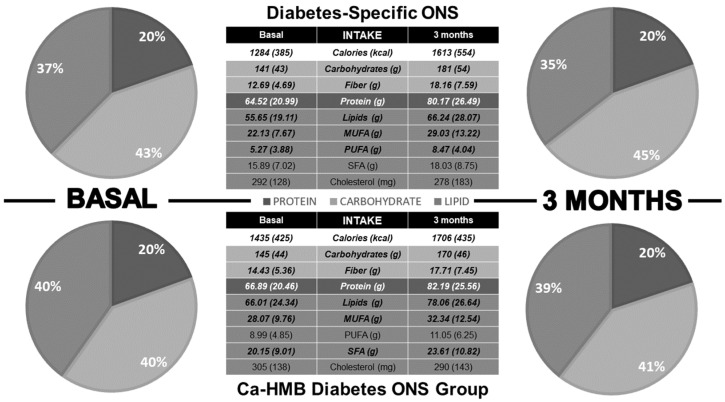
Dietary intake differences before and three months after initiation of medical nutritional therapy. Pie charts display the percentage contribution of macronutrients to total caloric intake, while tables present absolute values of macronutrients and the dietary lipid profile. Bold values indicate statistical significance (*p* < 0.05).

**Figure 4:**
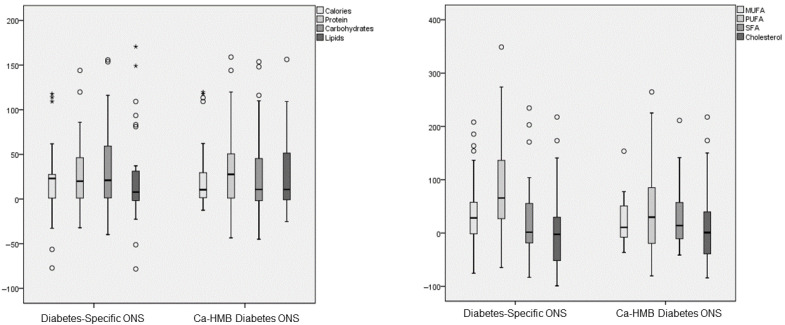
Differences in the percentage change of macronutrients and lipid dietary profile (monounsaturated fatty acids (MUFAs), polyunsaturated fatty acids (PUFAs), saturated fatty acids (SFTs), and cholesterol) parameters before and after the intervention in both groups (hydroxy-methyl-butyrate-enriched diabetes-specific oral nutritional supplementation (CaHMB Diabetes ONS) vs. Historic Cohort Supplements (Diabetes-Specific ONS). Circles: moderate outliers; stars: extreme outliers.

Three months after the start of the intervention, both groups showed an increase in caloric and macronutrient intake, although no changes were observed in TCV (Figure 2). In the Diabetes-Specific ONS group, there was an increase in MUFA and PUFA, with no increase in SFA or cholesterol. In contrast, the CaHMB Diabetes ONS group showed increases in MUFA, PUFA, and SFA (Figure 3). However, no significant differences were found between groups in terms of the magnitude of increase in macronutrient intake or lipid quality (Figure 4).

Regarding adherence, 48 patients (94.1%) in the Diabetes-Specific ONS group consumed 100% of the prescribed supplement; 1 patient (2%) consumed 50%, and 2 patients (3.9%) consumed 25%. In the CaHMB Diabetes ONS group, 36 patients (81.8%) consumed 100% of the prescribed supplement, while 4 patients (9.1%) consumed 75% and another 4 patients (9.1%) consumed 25%.

### 3.3. Changes in Nutritional Assessment Before and After Intervention

Patients in the CaHMB Diabetes ONS group showed increases in muscle mass parameters of the Rectus Femoris ultrasonography, specifically in RFMT and RFMA (Table 3). No significant differences were observed in muscle quality parameters (Table 3). In contrast, patients in the Diabetes-Specific ONS group demonstrated increases in arm and calf circumference, along with a decrease in RFMA (Table 3). No differences were found between the two groups in bioimpedanciometry or handgrip strength parameters (Table 3). There was a decrease in percentage weight loss in both groups, but there were no significant differences before and after intervention in the prevalence of malnutrition. There was a significant decrease in sarcopenia and dynapenia in both groups (Table 3).

**Figure 5:**
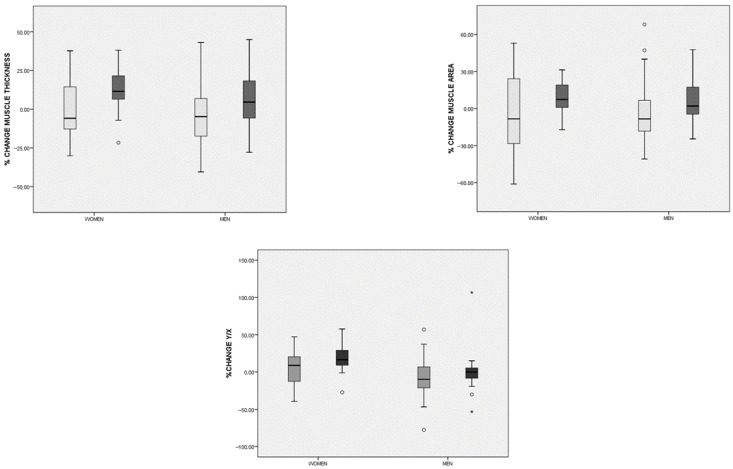
Differences in the percentage change in rectus femoris ultrasonography parameters before and after the intervention in both groups (hydroxy-methyl-butyrate-enriched diabetes-specific oral nutritional supplementation (CaHMB Diabetes ONS) (dark gray) vs. Historic Cohort Supplements (Diabetes-Specific ONS) (light gray)) based on gender. Circles: moderate outliers; stars: extreme outliers.

When comparing the percentage change in body composition parameters, significant differences emerged between the groups in muscle mass ultrasonography measures (Figure 5). The CaHMB group exhibited increases in RFMT, RFMA, and the Y/X index, whereas the Diabetes-Specific ONS group showed decreases in these parameters (Table 4).

Nutritional and inflammatory biochemical parameters did not differ between groups. Metabolic biochemical parameters showed changes only in the Diabetes-Specific ONS group, with an increase in HbA1c. No differences were observed in lipid profile (Table 5).

### 3.4. Relationship Between the Use of Hydroxy-Methyl-Butyrate-Enriched Diabetes-Specific Oral Nutritional Supplementation and Changes in Body Composition

In the multivariate analysis evaluating the likelihood of increases in rectus femoris ultrasonography parameters, a significant probability of increase in RFMT (above 0) was observed in patients who consumed an adapted diet along with CaHMB Diabetes ONS (regression coding: CaHMB Diabetes ONS = 1; Diabetes-Specific ONS = 0), with an odds ratio (OR) of 9.31 (95%CI: 2.16–40.131; *p* < 0.01) adjusted for gender, age, serum albumin, and HbA1c. Similarly, a higher probability of an increase in RFMA was noted in patients receiving CaHMB Diabetes ONS (OR = 3.96; 95%CI: 1.11–14.13; *p* = 0.03), also adjusted for gender, age, serum albumin, and HbA1c. However, no statistically significant results were found for the Y/X index in the multivariate analysis (OR = 2.78; 95%CI: 0.71–10.85; *p* = 0.03), when adjusted for the same covariates.

## 4. Discussion

This observational study, in adults with diabetes mellitus at risk of malnutrition, compares a historical cohort of patients treated with a diabetes-specific ONS with a cohort treated with a CaHMB-enriched diabetes-specific ONS. Although both groups achieved comparable improvements in overall dietary intake and reductions in sarcopenia prevalence, only the CaHMB-fortified intervention translated those gains into measurable enhancements in muscle architecture on ultrasound. These changes were an increase in muscle thickness and muscle area in the CaHMB group and a relative increase with respect to the control historic cohort in these parameters, and the relationship between anteroposterior and transversal axis in the cross-sectional image of QRF ultrasound. These effects remained robust after adjusting for sex, age, serum albumin, and glycaemic control.

Patients in this study exhibited BMI values at the upper end of the normal range, with normal brachial circumference and mildly reduced calf circumference. These findings are consistent with previous studies involving patients with DM, who often present signs of malnutrition despite having elevated BMI levels [3,14]. However, we did not observe elevated phase angle values in bioimpedance analysis, nor did we find low RFMT and RFMA values in muscle ultrasonography. The measurements were close to the diagnostic thresholds for sarcopenia, and comparable to those reported in the DRECO study (RFMT: 9.66 mm; RFMA: 3.66 cm^2^) [26]. These observations underscore the importance of a comprehensive nutritional assessment that includes dietary intake evaluation, body composition analysis, and muscle function testing [29]. No significant differences were found between groups in terms of body composition, muscle function parameters, or nutritional diagnosis. However, differences were observed in albumin and HbA1c levels. Additionally, a trend toward higher CRP levels in the experimental group may suggest a worse inflammatory status in these patients. Nevertheless, this did not produce differences in body composition parameters, as no significant variations were detected at that level [30].

Patients with DM often exhibit dietary changes that contribute to malnutrition, primarily due to persistent myths and inadequate nutritional education. These misconceptions frequently result in a carbohydrate intake that falls below the recommended percentage of total caloric value (%TCV), while the proportion of protein consumed tends to be relatively high, though not necessarily adequate in absolute terms [31]. Despite this, both groups in the study showed low overall intake of calories and protein. Macronutrient consumption increased across both groups, except for the experimental group, which showed an increase in saturated fatty acids but no corresponding rise in polyunsaturated fatty acids. When analyzing the consumption of ONS, there was a slight increase in volume in the CaHMB Diabetes ONS group relative to the Diabetes-Specific ONS group. A higher rate of treatment discontinuation was observed in the control group, which may have interfered with these results; nevertheless, these differences in supplement intake did not translate into significant changes in the oral diet itself, which supports the interpretation that CaHMB may have a distinct effect independent of dietary modification.

Both groups showed a reduction in weight loss percentage and a slight, non-significant increase in body weight. In the control group, arm and calf circumferences increased. These changes are likely related to increased energy intake and are consistent with findings from another study in patients treated with a diabetes-specific formula, which reported a reduction in percentage weight loss after three months of an adapted diet combined with a diabetes-specific ONS [14]. The use of CaHMB-enriched, hypercaloric, hyperproteic oral nutritional supplements (ONSs) in the NOURISH study led to an increase in body weight and an improvement in nutritional status, as assessed by the Subjective Global Assessment, compared to baseline in patients with disease-related malnutrition three months after hospital admission. However, this study did not include patients with DM [18]. Similarly, a study by Muangpaisan et al. reported an increase in BMI among patients treated with CaHMB-enriched ONS compared to a control group, along with an improvement in Malnutrition Universal Screening Tool (MUST) scores [32]. Additionally, Chew et al. showed an increase in weight, BMI, and mid-upper arm circumference during an intervention with an ONS containing CaHMB with respect to dietary counseling alone [33]. On the other hand, CaHMB supplementation has demonstrated benefits in increasing muscle mass and strength, as shown in a meta-analysis by Wen-Tao Gu et al., which evaluated five randomized trials in patients with sarcopenia [34].

The CaHMB Diabetes ONS group showed an increase in muscle mass parameters assessed by ultrasonography, specifically RFMT and RFMA, which was not observed in the control group. CaHMB-enriched ONS may help preserve muscle mass, improve recovery, and reduce complications. Evidence supports their use in both community and hospital settings as a therapeutic strategy to maintain muscle function [35]. Conversely, the study by De Luis et al. reported an increase in total muscle thickness (rectus femoris and vastus intermedius), but no significant change in the rectus femoris alone. These differences may be attributed to the duration of intervention, as their study lasted only three months [17]. A key distinction between these studies and ours lies in the patient population. Our cohort consisted of individuals with DM, the majority of whom also had oncologic conditions. The formula used in our study was a diabetes-specific CaHMB-enriched supplement, which differed from those used in other studies in terms of carbohydrate type and lipid profile, specifically, it was enriched in monounsaturated fats. These nutritional differences may influence baseline inflammation and its impact on muscle tissue. Another notable difference is our use of an active comparator from a historical cohort. Most studies evaluating muscle mass via ultrasound use a control group without ONS, where CaHMB is administered as a standalone supplement [36].

The use of AI in ultrasonographic image analysis can facilitate homogeneous segmentation of body composition compartments and enhance the evaluation of muscle mass and quality parameters. In this study, AI-based analysis proved effective in segmenting and detecting the percentage of muscle and fat within the muscle ROI. These quality parameters may help assess the impact of inflammatory burden on patients and monitor related changes. Muscle echogenicity can serve as a surrogate indicator of muscle inflammation in some diseases as inflammatory myopathies, but this fact may be challenging due to the multifaceted histopathological features [30]. Although no statistically significant differences in muscle quality changes were observed using this technique, there was a non-significant trend toward increased muscle percentage in the control group and a reduction in fat percentage in the experimental group. These findings contrast with those reported by De Luis et al., where a CaHMB-enriched supplement led to increased muscle percentage and decreased fat percentage in the ROI [17]. This discrepancy may be attributed to the baseline condition of the patients. In De Luis et. al.’s study, most participants did not have oncologic disease, unlike our sample [17]. This metabolic alteration of the muscle may mask the detection of changes in muscle quality, as observed in the study by Hong-Xiang et al., where the use of CaHMB-enriched ONS was associated with an increase in intramuscular fat, as assessed by AI-assisted ultrasonography [37]. In that study, three hypotheses were proposed to explain the increase in intramuscular fat: altered metabolism in patients with sarcopenia, short duration of nutrient exposure, and the presence of edema or inflammatory phenomena [37]. The similarity with our study lies in the unique muscular condition observed in patients with diabetes. Additionally, the duration of exposure to the intervention may have influenced the outcomes. Finally, the amount of BCAAs consumed could be a contributing factor.

Patients treated with CaHMB Diabetes ONS had poorer baseline glycemic control, but no changes were observed in this control after the intervention. In contrast, the group receiving a diabetes-specific ONS showed an increase in HbA1c following the intervention. This difference may be related to the fact that the diabetes-specific ONS group included more patients receiving insulin treatment, which could indicate a more advanced stage of diabetes mellitus. This outcome in diabetes-specific ONS may also be related to increased energy storage and the increase in carbohydrate consumption resulting from the diet, and the evidence of elevated albumin levels and increased arm and brachial circumferences, without a corresponding effect on muscle mass. Interestingly, patients treated with CaHMB Diabetes ONS showed a tendency toward reduced inflammatory parameters. Several studies have reported similar findings regarding the anti-inflammatory effects of CaHMB. For example, patients in pulmonary rehabilitation with non-cystic fibrosis bronchiectasis exhibited altered inflammatory markers and increased oxidative stress, while adding CaHMB-enriched ONS helped reduce neutrophil levels [38]. Likewise, a study by Nasimi et al. found that patients consuming a dairy product fortified with CaHMB and vitamin D experienced stabilization of CRP levels, whereas CRP values increased in the control group [39].

This study has several notable strengths. First, it is pioneering in comparing a diabetes-specific formula enriched with CaHMB against an active control supplement without CaHMB but matched in caloric and protein content, thereby allowing the specific effect of the metabolite to be isolated. Moreover, the use of AI-assisted muscle ultrasonography adds significant methodological value by enabling standardized segmentation and reducing inter-observer variability, thus improving the reliability of muscle mass and quality assessments. Another strength lies in the multidisciplinary follow-up involving both endocrinologists and dietitians, ensuring comprehensive metabolic and dietary monitoring across groups. Finally, the low attrition rate strengthens the internal validity of the findings, while the real-world clinical practice conditions enhance the external applicability of the results to everyday healthcare settings.

Nevertheless, the study also presents important limitations. Its observational design, including the use of a historical cohort as a control, restricts the ability to establish causality and may introduce selection bias. The relatively short intervention period (three months) limits the capacity to capture long-term changes in muscle mass, quality, and function. In addition, the study population was heterogeneous in terms of diabetes progression and treatment, with a high proportion of patients suffering from oncologic disease, which may have influenced metabolic responses and muscle outcomes, thus reducing the generalizability of the findings. A further limitation regarding the analysis of diet is that only adherence to the prescribed supplementation was prospectively recorded; dietary adherence was inferred from the nutrition survey rather than from direct monitoring of behavioral changes. Although changes in habitual eating patterns were not systematically documented, we evaluated shifts in macronutrient distribution, macronutrient quality, and the percentage contribution to total caloric intake to partially address this gap. Another limitation is that we did not collect data on angiotensin-converting enzyme (ACE) inhibitor (ACEi) or angiotensin receptor blocker (ARB) use, which may confound results because preclinical data show agents such as enalapril and losartan can modify body composition, reduce adiposity, and influence muscle strength and apoptotic pathways [40]. Future work should record and adjust for ACEi/ARB exposure or prospectively evaluate their interaction with CaHMB as a potential modifier of muscle mass and function. Finally, the lack of stratified analyses regarding different antidiabetic therapies and the limited assessment of muscle function parameters constrain the clinical interpretation of the results.

Future lines of investigation should focus on conducting randomized clinical trials with active comparators to better assess the effect of CaHMB in this patient population. Exploring its impact across different patient profiles, such as oncologic versus non-oncologic individuals, various types of diabetes, insulin use, or baseline CRP, could provide valuable insights into how these treatments influence muscle decline in distinct pathophysiological contexts. Additionally, longer intervention periods may offer a more comprehensive understanding of changes in muscle mass, quality, and function over time. Finally, studying patients at risk of malnutrition unrelated to disease, such as older adults, could help clarify the effects of this BCAA metabolite on muscle health in this vulnerable population.

## 5. Conclusions

In summary, supplementation with a diabetes-specific oral nutritional formula enriched with CaHMB led to significant improvements in muscle mass parameters, as assessed by AI-assisted ultrasonography, compared with a diabetes-specific formula without CaHMB. Although both groups achieved similar increases in overall dietary intake, only the CaHMB-enriched intervention translated these gains into measurable structural changes in muscle architecture, underscoring its anabolic potential. These findings support the role of targeted nutritional strategies in mitigating sarcopenia in patients with diabetes at risk of malnutrition, while highlighting the need for longer-term randomized controlled trials to confirm these results and further explore their functional and clinical implications.

## Figures and Tables

**Table 1:** Content of calories and macronutrients in hydroxy-methyl-butyrate-enriched diabetes-specific oral nutritional supplementation (CaHMB Diabetes ONS) vs. Historic Cohort Supplements (Diabetes-Specific ONS).

	CaHMB Diabetes ONS	Diabetes-Specific ONS
Caloric Content (kcal/100 mL)	163	120
Caloric Density (kcal/mL)	1.63	1.20
**Protein**		
Amount (g/100 mL)	8.32	6.6
%TCV	20	22
Hydroxy-methyl-butyrate (g/100 mL)	0.75	-
**Carbohydrates**		
Amount (g/100 mL)	12.75	12
%TCV	31	40
Sugars (g/100 mL)	0	2.5
Fiber (g/100 mL)	1.9	1.8
**Lipids**		
Amount (g/100 mL)	8.27	4.66
%TCV	45	35
Saturated (g/100 mL)	0.98	1.04
Monounsaturated (g/100 mL)	2.55	2.34
Polyunsaturated (g/100 mL)	1.65	1.12

%TCV: % total caloric value.

**Table 2:** Differences in nutritional assessment variables between groups (hydroxy-methyl-butyrate-enriched diabetes-specific oral nutritional supplementation (CaHMB Diabetes ONS) vs. Historic Cohort Supplements (Diabetes-Specific ONS).

	TOTAL (*n* = 95)	CaHMB Diabetes ONS (*n* = 44)	Diabetes-Specific ONS (*n* = 51)	*p*-Value
Age (years)	71.05 (10.67)	73.20 (8.73)	69.20 (11.86)	0.07
Sex (%M/%F)	56.8/43.2	61.4/38.6	52.9/47.1	0.41
**Anthropometry**				
BMI (kg/m^2^)	24.01 (4.91)	23.33 (4.57)	24.59 (5.16)	0.21
Weight Loss (%)	9.59 (3.40–16.14)	9.86 (1.57–15.69)	9.48 (3.74–16.31)	0.81
Arm circumference (cm)	25.38 (3.72)	25.22 (3.63)	25.51 (3.82)	0.71
Calf circumference (cm)	32.23 (3.98)	32.41 (4.47)	32.08 (3.54)	0.69
**Bioimpedanciometry**				
Resistance/height (ohm/m)	345.28 (69.92)	355.01 (75.65)	337.50 (64.70)	0.24
Reactance/height (ohm/m)	28.39 (6.36)	27.73 (6.69)	28.93 (6.09)	0.37
Phase Angle (°)	4.76 (0.91)	4.53 (0.90)	4.95 (0.87)	0.03
ASMI (kg/m^2^)	6.41 (1.21)	6.23 (1.22)	6.56 (1.19)	0.19
FFMI (kg/m^2^)	17.42 (2.77)	17.50 (2.82)	17.34 (2.76)	0.79
Total Water (%)	56.40 (6.22)	56.17 (6.27)	56.59 (6.23)	0.75
ECW/ICW	1.15 (0.40)	1.25 (0.52)	1.06 (0.25)	0.03
**Rectus Femoris Muscular Ultrasonography**				
SFT (cm)	0.59 (0.38–0.84)	0.45 (0.23–0.78)	0.66 (0.44–0.89)	0.16
RFMT (cm)	0.94 (0.25)	0.91 (0.21)	0.96 (0.28)	0.34
RFMA (cm^2^)	2.99 (0.93)	2.93 (0.76)	3.05 (1.05)	0.56
Y/X index	0.31 (0.21)	0.26 (0.21–0.32)	0.27 (0.22–0.36)	0.09
Mi (%)	45.41 (8.02)	44.90 (7.04)	45.80 (8.76)	0.59
FATi (%)	40.16 (5.57)	40.03 (5.02)	40.26 (6.02)	0.85
NMNFi (%)	14.44 (4.47)	15.07 (3.83)	13.94 (4.89)	0.23
Pennation Angle (°)	3.63 (2.26–5.49)	3.52 (2.27–5.41)	4.22 (2.16–5.59)	0.73
**Biochemical Parameters**				
Albumin (g/dL)	4.58 (1.29)	5.12 (1.69)	4.14 (0.54)	<0.01
Prealbumin (g/dL)	22.35 (7.36)	24.26 (7.27)	21.18 (7.25)	0.09
CRP (mg/dL)	2.99 (1–10.57)	3.66 (1–10.57)	2.65 (1.04–10.54)	0.82
CRP/albumin	0.60 (0.26–2.34)	0.74 (0.22–1.86)	0.59 (0.26–2.74)	0.39
CRP/prealbumin	0.12 (0.05–0.59)	0.10 (0.04–0.35)	0.12 (0.05–0.79)	0.16
Glucose (mg/dL)	125.45 (38.66)	125.05 (39.49)	125.80 (38.34)	0.93
HbA1c (%)	6.71 (1.46)	7.18 (1.68)	6.29 (1.08)	<0.01
**Nutritional Diagnosis**				
Sarcopenia (EWGSOP2) (%)	34.7	38.6	31.4	0.46
Dynapenia (EWGSOP2) (%)	53.7	61.4	58.8	0.80
Malnutrition (GLIM) (%)	82.1	86.4	78.4	0.31

ONS: oral nutritional supplement; BMI: body mass index; ASMI: Appendicular Skeletal Muscle Index; FFMI: fat-free mass index; ECW/ICW: extracellular–intracellular water index; SFT: subcutaneous fat thickness; RFMT: rectus femoris muscle thickness; RFMA: rectus femoris muscle area; Mi: muscle index; FATi: fat index; NMNFi: no muscle, no fat index; CRP: C-reactive protein; HbA1c: glycated hemoglobin; EWGSOP2: European Working Group on Sarcopenia in Older People; GLIM: Global Leadership Initiative on Malnutrition.

**Table 3:** Changes in nutritional assessment before and 3 months after intervention in both groups (hydroxy-methyl-butyrate-enriched diabetes-specific oral nutritional supplementation (CaHMB Diabetes ONS) vs. Historic Cohort Supplements (Diabetes-Specific ONS)).

	CaHMB Diabetes ONS			Diabetes-Specific ONS		
	Start (*n* = 44)	3 Months (*n* = 44)	*p*-Value	Start (*n* = 51)	3 Months (*n* = 51)	*p*-Value
**Anthropometry**						
BMI (kg/m^2^)	23.33 (4.57)	23.54 (3.85)	0.43	24.59 (5.16)	24.79 (5.21)	0.29
%Weight Loss	−11.39 (11.57)	+1.50 (6.85)	<0.01	10.88 (9.03)	+1.24 (4.80)	<0.01
Arm circumference (cm)	25.23 (3.67)	25.38 (2.70)	0.63	25.51 (3.82)	25.99 (3.87)	0.03
Calf circumference (cm)	32.42 (4.52)	31.96 (3.61)	0.23	32.08 (3.54)	32.75 (3.65)	0.03
**Bioimpedanciometry**						
Resistance/height (ohm/m)	352.33 (71.34)	345.26 (70.15)	0.32	335.44 (63.69)	333.54 (67.06)	0.68
Reactance/height (ohm/m)	27.92 (6.83)	27.85 (7.59)	0.96	28.68 (5.88)	28.81 (6.72)	0.87
Phase Angle (°)	4.55 (0.80)	4.56 (0.71)	0.96	4.94 (0.88)	4.98 (0.98)	0.72
ASMI (kg/m^2^)	6.23 (1.20)	6.33 (1.05)	0.47	6.59 (1.18)	6.66 (1.22)	0.28
FFMI (kg/m^2^)	17.51 (2.79)	17.54 (1.96)	0.89	17.41 (2.74)	17.65 (2.86)	0.21
Total Water (%)	56.66 (6.44)	56.31 (6.43)	0.61	56.46 (6.23)	56.37 (6.42)	0.83
ECW/ICW	1.21 (0.38)	1.17 (0.22)	0.56	1.06 (0.26)	1.07 (0.22)	0.87
**Rectus Femoris Muscular Ultrasonography**						
SFT (cm)	0.54 (0.07)	0.56 (0.06)	0.56	0.73 (0.41)	0.75 (0.44)	0.41
RFMT (cm)	0.92 (0.21)	1.04 (0.31)	0.02	0.95 (0.29)	0.89 (0.28)	0.09
RFMA (cm^2^)	2.98 (0.74)	3.37 (1.20)	<0.01	3.04 (1.06)	2.78 (1.15)	<0.05
Y/X index	0.27 (0.07)	0.29 (0.09)	0.11	0.35 (0.28)	0.29 (0.16)	0.08
Mi (%)	45.07 (6.78)	45.31 (6.64)	0.87	45.76 (8.87)	45.73 (6.99)	0.99
FATi (%)	40.09 (5.04)	39.93 (5.16)	0.89	40.30 (6.11)	40.30 (4.86)	0.98
NMNFi (%)	14.84 (3.69)	14.76 (3.02)	0.87	13.94 (4.95)	13.96 (4.58)	0.98
Pennation Angle (°)	3.93 (2.49)	4.43 (2.50)	0.28	4.11 (2.33)	4.05 (2.76)	0.91
**Muscle Strength**						
Handgrip Strength (kg)	20.36 (11.04)	20.68 (11.33)	0.51	20.72 (8.87)	21.55 (8.85)	0.07
**Nutritional Diagnosis**						
Sarcopenia (EWGSOP2) (%)	38.6	34.1	<0.01	31.4	29.4	<0.01
Dynapenia (EWGSOP2) (%)	61.4	56.8	<0.01	58.8	51	<0.01
Malnutrition (GLIM) (%)	86.4	61.4	0.54	78.4	49	0.10

ONS: oral nutritional supplement; BMI: body mass index; ASMI: Appendicular Skeletal Muscle Index; FFMI: fat-free mass index; ECW/ICW: extracellular–intracellular water index; SFT: subcutaneous fat thickness; RFMT: rectus femoris muscle thickness; RFMA: rectus femoris muscle area; Mi: muscle index; FATi: fat index; NMNFi: no muscle, no fat index; EWGSOP2: European Working Group on Sarcopenia in Older People; GLIM: Global Leadership Initiative on Malnutrition.

**Table 4:** Differences in the percentage of change in nutritional variables before and 3 months after intervention between groups. (hydroxy-methyl-butyrate-enriched diabetes-specific oral nutritional supplementation (CaHMB Diabetes ONS) vs. Historic Cohort Supplements (Diabetes-Specific ONS).

	CaHMB Diabetes ONS (*n* = 44)	Diabetes-Specific ONS (*n* = 51)	*p*-Value
% Δ weight	+1.97 (−1.96 ± 5.32)	+ 1.08 (−2.12 ± 4.55)	0.61
% Δ Arm circumference	0 (−2.33 ± 6.33)	+1.33 (0 ± 4.92)	0.79
% Δ Calf circumference	0 (−4.05 ± 3.70)	+1.69 (0 ± 3.44)	0.11
% Δ Resistance	−0.47 (−5.46 ± 10.07)	−1.52 (−6.06 ± 4.57)	0.84
% Δ Reactance	−1.69 (−9.51 ± 14.42)	0 (−8.99 ± 11.76)	0.81
% Δ Phase Angle	+2.13 (−11.36 ± 11.43)	0 (−8.99 ± 11.76)	0.79
% Δ ASMI	+2.49 (−3.59 ± 6.59)	+1.41 (−1.51 ± 4.68)	0.59
% Δ FFMI	+2.22 (−3.03 ± 5.45)	+2.15 (−2.44 ± 4.64)	0.94
% Δ Total Water	−0.28 (−5.71 ± 3.08)	−0.14 (−3.49 ± 2.32)	0.58
% Δ ECW/ICW	−2.35 (−11.05 ± 14.03)	−0.87 (−8.42 ± 8.11)	0.62
% Δ SFT	+5.83 (−4.79 ± 26.89)	+0.75 (−14.73 ± 22.03)	0.31
% Δ RFMT	+9.17 (−4.40 ± 21.05)	−6.30 (−18.57 ± 12.56)	<0.01
% Δ RFMA	+5.84 (−3.33 ± 21.58)	−9.34 (−25.78 ± 12.02)	<0.01
% Δ Y/X	+4.33 (−7.74 ± 14.53)	−3.06 (−18.61 ± 17.29)	0.03
% Δ Mi	−1.01 (−15.23 ± 14.53)	+1.69 (−12.06 ± 16.21)	0.75
% Δ FATi	−2.41 (−11.82 ± 7.98)	+1.09 (−9.22 ± 8.21)	0.67
% Δ NMNFi	+3.41 (−19.69 ± 17.84)	−0.11 (−28.59 ± 34.85)	0.82
% Δ Handgrip Strength	0 (−6.30 ± 12.95)	0 (−0.66 ± 14.56)	0.59

ONS: oral nutritional supplement; ASMI: Appendicular Skeletal Muscle Index; FFMI: fat-free mass Index; ECW/ICW: extracellular–intracellular water index; SFT: subcutaneous fat thickness; RFMT: rectus femoris muscle thickness; RFMA: rectus femoris muscle area; Mi: muscle index; FATi: fat index; NMNFi: no muscle, no fat index.

**Table 5:** Differences in biochemical parameters before and after the intervention in both groups (hydroxy-methyl-butyrate-enriched diabetes-specific oral nutritional supplementation (CaHMB Diabetes ONS) vs. Historic Cohort Supplements (Diabetes-Specific ONS)).

	CaHMB Diabetes ONS			Diabetes-Specific ONS		
	Baseline (*n* = 44)	3 Months (*n* = 44)	*p*-Value	Baseline (*n* = 51)	3 Months (*n* = 51)	*p*-Value
HbA1c (%)	7.37 (1.77)	7.52 (1.84)	0.18	6.19 (1.12)	7.17 (1.13)	<0.01
Glucose (mg/dL)	125.16 (41.49)	125.39 (37.64)	0.97	124.87 (38.79)	125.81 (29.56)	0.83
Total cholesterol (mg/dL)	177.60 (51.89)	171.66 (46.36)	0.51	160.69 (44.07)	162.56 (41.16)	0.68
HDL cholesterol (mg/dL)	58.59 (23.47)	62.29 (27.03)	0.31	57.87 (29.79)	59.98 (24.21)	0.25
LDL cholesterol (mg/dL)	95.68 (44.89)	92.18 (35.48)	0.69	83.93 (35.39)	85.64 (33.26)	0.66
Triglycerides (mg/dL)	108.23 (54.76)	94.69 (40.67)	0.07	116 (64.51)	110.12 (53.33)	0.24
Albumin (g/dL)	5.23 (1.78)	5.12 (1.74)	0.52	4.13 (0.51)	4.26 (0.41)	0.02
Prealbumin (g/dL)	22.03 (7.19)	22.53 (6.89)	0.27	21.21 (7.07)	22.66 (6.74)	0.04
CRP (mg/dL)	3.66 (1–10.57)	1.98 (1–5.20)	0.69	2.65 (1.04–10.54)	2.10 (1–5.28)	0.62
CRP/albumin	0.74 (0.22–1.87)	0.45 (0.22–1.14)	0.72	0.59 (0.26–2.74)	0.53 (0.24–1.33)	0.31
CRP/prealbumin	0.10 (0.04–0.35)	0.11 (0.05–0.27)	0.16	0.12 (0.05–0.79)	0.07 (0.05–0.19)	0.34

CRP: C-reactive protein; HBA1c: glycated hemoglobin.

## Data Availability

The data presented in this study are available upon request from the corresponding author due to ethical reasons.
